# An Insight into Kiwiberry Leaf Valorization: Phenolic Composition, Bioactivity and Health Benefits

**DOI:** 10.3390/molecules26082314

**Published:** 2021-04-16

**Authors:** Ana Margarida Silva, Diana Pinto, Iva Fernandes, Victor de Freitas, María de la Luz Cádiz-Gurrea, Paulo Costa, Cristina Delerue-Matos, Francisca Rodrigues

**Affiliations:** 1 REQUIMTE/LAQV-Polytechnic of Porto, School of Engineering, Rua Dr. António Bernardino de Almeida, 431, 4249-015 Porto, Portugal; ana.silva@graq.isep.ipp.pt (A.M.S.); diana.pinto@graq.isep.ipp.pt (D.P.); maria.gurrea@graq.isep.ipp.pt (M.d.l.L.C.-G.); cmm@isep.ipp.pt (C.D.-M.); 2 REQUIMTE/LAQV, Department of Chemistry and Biochemistry, Faculty of Sciences, University of Porto, Rua do Campo Alegre, s/n, 4169-007 Porto, Portugal; iva.fernandes@fc.up.pt (I.F.); vfreitas@fc.up.pt (V.d.F.); 3 REQUIMTE/UCIBIO, MedTech-Laboratory of Pharmaceutical Technology, Department of Drug Sciences, Faculty of Pharmacy, University of Porto, Rua de Jorge Viterbo Ferreira, 228, 4050-313 Porto, Portugal; pccosta@ff.up.pt

**Keywords:** kiwiberry leaf, microwave-assisted extraction, phenolic profile, biological activities

## Abstract

During kiwiberry production, different by-products are generated, including leaves that are removed to increase the fruit’s solar exposure. The aim of this work was to extract bioactive compounds from kiwiberry leaf by employing microwave-assisted extraction (MAE). Compatible food solvents (water and ethanol) were employed. The alcoholic extract contained the highest phenolic and flavonoid contents (629.48 mg of gallic acid equivalents (GAE) per gram of plant material on dry weight (dw) (GAE/g dw) and 136.81 mg of catechin equivalents per gram of plant material on dw (CAE/g dw), respectively). Oppositely, the hydroalcoholic extract achieved the highest antioxidant activity and scavenging activity against reactive oxygen and nitrogen species (IC_50_ = 29.10 μg/mL for O_2_^•−^, IC_50_ = 1.87 μg/mL for HOCl and IC_50_ = 1.18 μg/mL for ^•^NO). The phenolic profile showed the presence of caffeoylquinic acids, proanthocyanidin, and quercetin in all samples. However, caffeoylquinic acids and quercetin were detected in higher amounts in the alcoholic extract, while proanthocyanidins were prevalent in the hydroalcoholic extract. No adverse effects were observed on Caco-2 viability, while the highest concentration (1000 µg/mL) of hydroalcoholic and alcoholic extracts conducted to a decrease of HT29-MTX viability. These results highlight the MAE potentialities to extract bioactive compounds from kiwiberry leaf.

## 1. Introduction

Kiwiberry (KB; *Actinidia arguta*) is a delicate, small grape-sized fruit, characterized by the absence of hair and the presence of a pleasant aroma and flavor [1]. According to different authors, KB is an excellent source of vitamin C, phenolic compounds (tannins and flavanols), organic acids (citric, quinic, and malic acids), sugars (sucrose, glucose and fructose), minerals (potassium, calcium, magnesium, copper, iron, and manganese), and carotenoids (lutein and β-carotene), being associated with multiple benefits for the consumer’s well-being [1,2]. Indeed, when incorporated in the diet, KB might prevent diseases caused by the activity of free radicals, such as diabetes or neurogenerative diseases, representing an important natural antioxidant source [2,3].

Worldwide, the production of KB is increasing mainly due to the biological properties reported (such as antioxidants) which allow its classification as a superfood [4]. The most recent data stated that in 2015/16 the worldwide production was almost 1600 tons [5]. In Portugal, the national production achieved a record value of 120 tons in 2016 [5]. Nevertheless, during KB production different by-products are generated, such as fruits without caliber to be commercialized, pomace, or leaves [3,6]. According to Marangi et al., the leaf remotion during production increases the exposure of fruits to the sun [6]. However, to the best of our knowledge, the information about them is scarce. Cyboran et al. reported that KB leaf is a source of polyphenolic compounds, such as neochlorogenic, chlorogenic, and cryptochlorogenic acids, as well as catechin and flavonols [7]. The authors also showed that KB leaf can protect red blood cell membranes from oxidation induced by ultraviolet (UV) radiation [7]. Recently, Almeida et al. employed three different solvents (ethanol, water, and ethanol-water) to extract by conventional techniques to the KB leaf, reporting that the alcoholic extract exhibited the highest antioxidant activity, as well as the total phenolic and flavonoid contents (TPC and TFC, respectively) [8]. According to the authors, the phenolic composition revealed that flavonoids and phenolic acids are the predominant bioactive compounds [8]. Nevertheless, few studies are available describing the biological activities of KB leaf extracts. According to them, the extracts may improve the glucose tolerance, potentiating the inhibitory capacity of α-glucosidase, and be benefic for the treatment and prevention of obesity and cognitive dysfunctions in diabetes [9,10]. Furthermore, anti-inflammatory and antimicrobial activities were described [8,11].

Nevertheless, extraction is the most limiting step during this process [12]. Conventional extraction methodologies have been the most used procedures [3,8]. Nonetheless, these techniques have different disadvantages, such as time-consuming extraction, employment of elevated temperatures, and high amounts of solvents, as well as low extraction yields [13,14]. Due to these drawbacks, the implementation of green extraction techniques arises as a challenge. Microwave-assisted extraction (MAE) is an environmentally friendly technique, being considered one of the best methods to extract phenolic compounds due to its high extraction rate, short extraction time, and better product quality [15,16]. The MAE efficiency is due to faster energy absorption and quicker and selective heating, which reduces the thermal gradients and improves the extraction yield [17,18]. The main advantage of this technique is the facility of the solvent to penetrate inside the cell walls, breaking the phenolics and cell walls linkages, which leads to a high phenolic compounds recovery [12].

The aim of this work was to evaluate the KB leaf bioactive compounds recovered by a green and environmentally friendly extraction technique (MAE) in order to valorize this by-product for other fields, such as the food industry. With this purpose, the extracts were screened and characterized regarding phenolic compounds, radical scavenging activity, antioxidant potential, and effects on intestinal cell viability. To the best of our knowledge, this is the first study that reports the use of MAE on KB leaf.

## 2. Results and Discussion

### 2.1. Phenolic Content and In Vitro Antioxidant/Antiradical Activities

Polyphenols are a vast group of compounds, being responsible for the antioxidant activity of plant extracts due to their properties such as redox potential or capacity to chelate metals [19]. Table 1 summarizes the total polyphenol content (TPC), total flavonoid content (TFC), and in vitro antioxidant/antiradical activities obtained for the different extracts.

According to Table 1, the TPC ranged between 120.99 mg of gallic acid equivalents (GAE) per gram of plant material on dry weight (dw) (mg GAE/g dw) and 629.48 mg GAE/g dw, with the highest value corresponding to the alcoholic extract and the lowest to the aqueous one. Significant differences (*p* < 0.05) were observed between the different extracts, probably due to the solubility of the solvents. The values obtained for the hydroalcoholic and alcoholic extracts were higher than the ones reported by Almeida et al. [8] that evaluated the phenolic content of KB leaf using a conventional methodology (140.72 mg GAE/g and 440.71 mg GAE/g for the hydroalcoholic and alcoholic extracts, respectively).

In what concerns to the TFC, the aqueous extract presented the lowest result (62.17 mg of catechin equivalents (CAE) per gram of plant material on dw (mg CAE/g dw)), followed by the hydroalcoholic (114.20 mg CAE/g dw) and the alcoholic (136.81 mg CAE/g dw) ones (Table 1), with significant differences between samples (*p* < 0.001). Almeida et al. reported that the TFC of KB leaf extracted by maceration with water and ethanol were, respectively, 136.72 mg CAE/g dw and 318.11 mg CAE/g dw [8]. These values were higher than the ones reported in the present study, probably due to the higher percentages of ethanol, which decrease the dielectric constant of the mixture and the capacity to absorb energy [20]. Although the increase of extraction time led to an improvement of the extraction yield, the longer application of microwave may conduct to the phenolic compounds degradation [20].

According to Table 1, the hydroalcoholic extract exhibited the best result in the FRAP assay (3079.94 μmol FSE/g dw), while the worst was achieved by the aqueous extract (1801.98 μmol FSE/g dw). No significant differences (*p* = 0.988) were observed between the alcoholic and hydroalcoholic extracts. These results are in line with the value reported by Almeida et al. for the hydroalcoholic extract obtained by conventional extraction (3076.35 FSE/g dw) [8].

The scavenging activity on the DPPH and ABTS radicals were also evaluated (Table 1). Regarding the DPPH radical scavenging, the hydroalcoholic extract showed the lowest IC_50_ (95.22 μg/mL), oppositely to the aqueous extract that presented the highest value (211.14 μg/mL). Once again, no significant differences (*p* = 0.970) were observed between the hydroalcoholic and alcoholic extracts. The IC_50_ obtained in this study were lower than the one reported by Marangi et al. for the KB leaf extracted with Multi-Frequency Multimode Modulated Technology (MMM) (270.17 μg/mL) [6]. However, Almeida et al. reported an IC_50_ of 53.95 μg/mL for the alcoholic extract, while for the hydroalcoholic extract the IC_50_ was 1097.28 μg/mL [8].

Regarding the ABTS^•+^ scavenging activity, the different samples showed effective scavenging activity as reported in Table 1. The IC_50_ was 131.58 μg/mL, 142.96 μg/mL, and 219.14 μg/mL for the hydroalcoholic, alcoholic, and aqueous extracts, respectively. Significant differences (*p* < 0.05) were observed between the aqueous and hydroalcoholic extracts, as well as between the aqueous and the alcoholic ones. On the contrary, no significant differences (*p* = 0.321) were reported between the hydroalcoholic and alcoholic extracts.

### 2.2. Phenolic Profile of Kiwiberry Leaf Extracts

Powerful and advanced techniques have been used to carry out a comprehensive characterization of the polar profile from KB leaf extracts. To the best of our knowledge, this is the first deep identification of polar compounds obtained from KB leaf by MAE.

**Figure 1:**
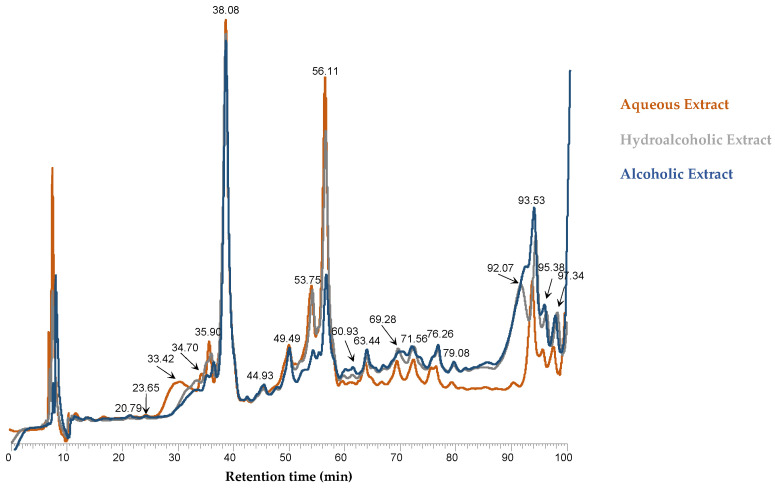
Chromatographic profile of phenolic compounds obtained by HPLC-DAD-MS (280 nm) of aqueous, hydroalcoholic, and alcoholic extracts of KB leaf extracts.

Figure 1 presents the phenolic profile obtained for the aqueous (a), hydroalcoholic (b) and alcoholic (c) extracts.

Moreover, Table 2 summarizes the different phenolic compounds identified, as well as the relative percentage of each constituent expressed as a percent of dry plant weight in the different samples.

As reported in Table 2, the qualitative phenolic profile is similar for the different extracts, being the differences related to the amount of each class. In a general way, the most representative groups in KB leaf extracts are phenolic acids, followed by flavonoids (derivatives of flavan-3-ol and flavonols). Caffeoylquinic acids, apigenin-rutinoside, and quercetin-pentoside-hexoside are present in all samples. The mass at *m/z* [M − H]^−^ 707.51 detected for caffeoylquinic acids at 38 min and 56 min should correspond to adducts formed at mass source due to its high concentration. Indeed, in the base of the peak, the mass obtain is 353.51 corresponding to the caffeoylquinic acid molecule.

Proanthocyanidins are also present in all samples, similarly to quercetin-*O*-deoxyhexoside-rutinoside and quercetin-*O*-rutinoside/quercetin-*O*-galactoside-*O*-rhamnoside/quercetin-*O*-rhamnosyl-galactoside.

Quantitatively, the principal phenolic compounds in all extracts were caffeoylquinic acids, with the alcoholic extract achieving the highest relative amount (35.33%). Regarding apigenin-rutinoside, the aqueous extract showed the maximum relative amount (15.07%) and the alcoholic the lowest (8.03%). The main flavonoids quantified in all extracts were proanthocyanidins, showing the total highest amount in the hydroalcoholic extract (12.61%). The alcoholic extract was particularly rich in quercetin-*O*-deoxyhexoside-rutinoside and quercetin-*O*-rutinoside/quercetin-*O*-galactoside-*O*-rhamnoside/quercetin-*O*-rhamnosyl-galactoside (18.46 and 11.06%, respectively).

Our results are in line with Marangi et al. and Almeida et al. [6,8]. Marangi et al. studied the phenolic profile of KB leaf extracted by MMM through HPLC-DAD-EIS-MS [6]. The authors identified and quantified not only flavonoid derivatives, but also chlorogenic acids (particularly 3/5-caffeoylquinic acids and quinic acid) and gallic acid derivatives (namely kaempferol-3-*O*-(acetyl-rhamnoside)-hexoside and quercetin-3-*O*-(acetyl-rhamnoside)-hexoside). The major compounds present were flavonoid derivatives (6.30%), as this value lower than the one reported in the present study. Almeida et al. also identified and quantified 24 phenolic compounds in KB leaf extracted by conventional methodologies [8]. According to the authors, hydroxycinnamic acids, such as neochlorogenic acid and caffeic acid derivatives, are particularly present in the aqueous extract, while flavan-3-ols (namely catechin and derivatives) are the majority of compounds in the alcoholic extract.

### 2.3. Reactive Species Scavenging Capacity

The reactive species production is a natural bioprocess that occurs during cell signaling or as a defense against infectious agents [8]. Oxidative stress is the result of an imbalance between the pro-oxidant reactive species production and the cells antioxidant defense. This condition is responsible for the damage of the major cellular components, such as DNA, proteins or lipids [3]. Phenolic compounds can counterbalance these reactive species. Table 3 summarizes the scavenging capacity of KB leaf extracts against reactive oxygen and nitrogen species (ROS and RNS, respectively).

Catalase and superoxide dismutase are enzymes responsible for the dismutation of O_2_^•−^, a precursor of a diversity of powerful oxidants. The scavenge of this species prevents different pathophysiological situations, such as inflammation, hypertension, or atherosclerosis [3]. As exposed in Table 3, the IC_50_ values of the KB leaf extracts scavenge capacity ranged between 29.10 μg/mL and 61.50 μg/mL for the hydroalcoholic and aqueous extracts, respectively. The hydroalcoholic extract achieved an IC_50_ considerably inferior to the one described for the positive control (catechin). No significant differences (*p* = 0.930) were observed between the hydroalcoholic and alcoholic extracts. In addition, the aqueous extract and positive controls had significant differences (*p* < 0.001) for all samples. The results of the hydroalcoholic and alcoholic extracts were in line with the values obtained by Almeida et al. that reported IC_50_ between 24.44 and 36.46 μg/mL, namely for the aqueous and alcoholic extracts [8].

HOCl is another oxygen species that reacts with biomolecules, mainly during the inflammatory processes. This species is formed in neutrophils as a result of the reaction between peroxide hydrogen and chloride ions, being catalyzed by myeloperoxidase enzyme [19]. According to Table 3, the hydroalcoholic extract presented a good capacity to scavenge HOCl (IC_50_ = 1.87 μg/mL), followed by the aqueous and alcoholic extracts (IC_50_ = 1.94 μg/mL and IC_50_ = 2.08 μg/mL, respectively). Significant differences were observed between the three extracts and catechin (*p* < 0.001). In addition, no significant differences (*p* > 0.05) were perceived between all samples, as well as between the gallic acid and the aqueous and hydroalcoholic extracts. Once again, the scavenging capacity obtained was higher than the one reported by Almeida et al. for this species [8]. On the contrary, the IC_50_ value reported by Marangi et al. using MMM technology to extract KB leaf was in line with the present study [6].

The ORAC assay is a fast and simple methodology to evaluate the scavenging of ROO^•^ by the extracts. The results demonstrated that the aqueous extract was the most effective, while the alcoholic and hydroalcoholic extracts were less successful (Table 3). No differences (*p* = 1.00) were perceived among the different extracts. On the contrary, significant differences (*p* < 0.05) were reported between catechin, gallic acid, and the extracts. Marangi et al. also evaluated the scavenging activity of the MMM KB leaf extracts against this species, reporting a lower value (0.00051) [6].

The scavenging activity of ^•^NO was also assessed. This reactive species is produced in vivo by nitric oxide synthase, which may occur in three isoforms: neuronal nitric oxide synthase (nNOS), inducible nitric oxide synthase (iNOS) and endothelium nitric oxide synthase (ecNOS) [21]. The first and third enzymes are considered constitutive forms and can be found in cells and activated by calcium [21]. The second isoform is expressed after exposure to specific combinations of cytokines [21]. In the presence of this enzyme, the nitrosation, nitration, and oxidation processes are increased [21], leading to the formation of toxic oxidants and nitrating agents that may result in numerous disorders, such as diabetes, cardiovascular, and neurodegenerative diseases and cancer [22]. According to Table 3, the hydroalcoholic extract showed the maximum scavenging efficacy against ^•^NO, while the alcoholic extract had the lowest (IC_50_ = 1.18 μg/mL and IC_50_ = 1.74 μg/mL, respectively). No significant differences between the extracts were observed (*p* > 0.05). Comparatively with Almeida et al. that used conventional extraction techniques (IC_50_ = 0.83 μg/mL and IC_50_ = 1.75 for the alcoholic and hydroalcoholic extracts, respectively), the scavenging activity achieved in our study was higher [8]. The same occurs regarding Marangi et al. that obtained the highest IC_50_ (IC_50_ = 3.80 μg/mL) [6].

In a general way, the results obtained for ROS and RNS scavenging activities (Table 3) are in line with the ones achieved for DPPH, ABTS and FRAP assays (Table 1), with the hydroalcoholic extract demonstrating the best results. In fact, radical scavenging assays are extremely important since they employ reactive species that exists in the human body, mimetizing the in vivo effects.

### 2.4. Cytotoxic Effects of Kiwiberry Leaf Extracts towards Intestinal Cells

**Figure 2:**
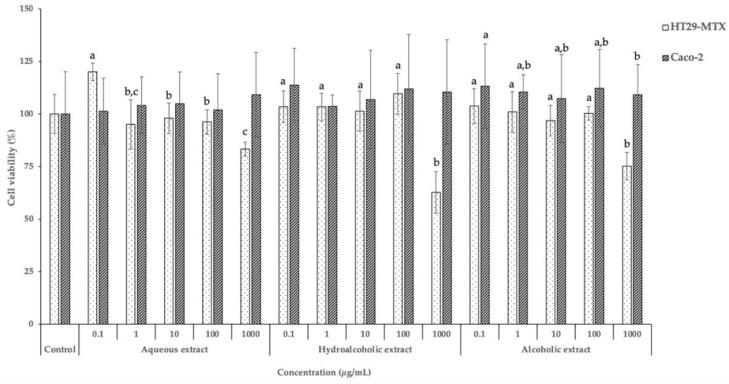
Effect of KB leaf extracts, at different concentrations, on the viability of HT29-MTX and Caco-2 cells, measured by an MTT assay (*n* = 3). Different letters (a, b, c) mean significant differences between concentrations of the same sample (*p* < 0.05).

The effect of KB leaf extracts on intestinal cell lines were evaluated by a 3-(4,5-dimethylthiazol-2-yl)-2,5-diphenyltetrazolium bromide (MTT) assay, as shown in Figure 2. Caco-2 and HT29-MTX are intestinal cell lines commonly selected to mimic the absorption processes of bioactive compounds [23].

According to Figure 2, the three extracts did not lead to a decrease of Caco-2 viability after exposure to the different concentrations, presenting results around 100%.

Oppositely, the viability of HT29-MTX decreased after exposure to the highest concentration of the hydroalcoholic and alcoholic samples, displaying viabilities of 62.68% and 75.16%, respectively. Indeed, at the highest concentration, the aqueous extract did not decrease the HT29‑MTX viability, without significant differences (*p* > 0.05) between the concentrations of 1.0, 10.0, and 100.0 µg/mL. Nevertheless, the concentration of 0.1 μg/mL was significantly different (*p* < 0.05), achieving a viability above 100%. These differences could be related with the bioactive compounds present in each extract. According to Table 2, the hydroalcoholic and alcoholic extracts presented the highest amounts of bioactive compounds. Specifically, the hydroalcoholic extract was rich in flavonoids, mainly proanthocyanidins (12.61%), while the alcoholic extract exhibited high amounts of caffeoylquinic acids and quercetin (35.33% and 29.52%, respectively). These phenolic compounds present in high concentrations are probably responsible for the toxic effects observed.

Almeida et al. also evaluated the effects of KB leaf conventional extracts on the viability of Caco-2 and HT29-MTX cell lines, using the same range of concentrations [8]. The authors did not observe a decrease of HT29-MTX viability for all tested concentrations, while for Caco-2 the viability decreased to 58% and 60%, for the hydroalcoholic and alcoholic extracts, respectively, after exposure to the highest concentration (1000 µg/mL) [8]. These differences are probably related with the extraction technique employed by the authors. Conventional methodologies, such as maceration, are normally associated with low bioactive compounds extraction when compared with MAE. This normally occurs because microwaves are capable of penetrating the sample and interacting with polar components, allowing a high extraction efficiency of bioactive compounds.

### 2.5. Enzymatic Activity

**Figure 3:**
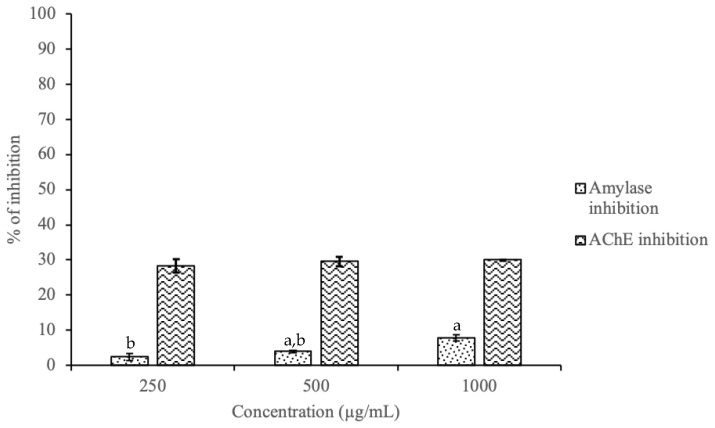
Inhibitory activities of hydroalcoholic extract of KB leaf against *α*-amylase and AChE activity (*n* = 3). Different letters (a, b) mean significant differences between different concentrations (*p <* 0.05).

*α*-Amylase is an enzyme found in saliva, which has an important role in the initial breakdown of food, particularly starch and glycogen [24]. In the small intestine, *α*-amylase regulates the postprandial glucose levels, decreasing the absorption of glucose [25]. The determination of *α*-amylase activity is normally employed to evaluate the interaction between polyphenols and proteins [26]. Polyphenols have good inhibitory activity on *α*-amylase, decreasing hyperglycemia and preventing diseases, such as diabetes and obesity [27]. Considering the promising composition of the hydroalcoholic KB leaf extracts, the potential inhibitory activity of *α*-amylase by this extract was evaluated (Figure 3). The maximum inhibition achieved was 7.73% and the minimum 2.34% for 1000 and 250 μg/mL, respectively. No significant differences (*p* > 0.05) were observed between the concentration of 250 and 500 μg/mL. These results are lower than the ones obtained by Wang et al. for *Camelia sinensis* peel extracts prepared by conventional methods [25]. According to the authors, the best inhibitory effect was achieved with 600 μg/mL (highest concentration tested) and the highest effect was found in the butanol fraction while the lower was in the water fraction (38.3% and 8.24%, respectively) [25]. These differences may be due to the solvents employed, since the *C. sinensis* extracts enriched with butanol, ethyl acetate, and chloroform fractions presented mild inhibition of *α*-amylase. Nevertheless, despite the low results obtained, to the best of our knowledge this is the first study that evaluated the inhibitory activity of *α*-amylase for KB leaf, it being necessary to screen other solvents in the future.

AChE is an enzyme responsible for the hydrolysis of acetylcholine into choline and acetic acid [28], exerting an extremely important function in the central and peripheral nervous systems. The AChE inhibitors have beneficial effects on human memory, being used in the treatment of diseases such as Alzheimer’s [29]. Considering the results reported in the previous sections, the hydroalcoholic extract was selected for this assay. The AChE inhibitory activity ranged between 28.30% and 30.00%, with the highest value corresponding to the concentration of 1000 μg/mL and the lowest to 250 μg/mL (Figure 3). No significant differences (*p* > 0.05) were observed between the different concentrations. Moyo et al. also evaluated the AChE inhibitory activity of *Sclerocarya birrea* young stems and *Harpephyllum caffrum* leaf extracts employing different solvents (petroleum ether, dichloromethane, and 50% (*v/v*) methanol) [30]. The highest percentage inhibition (38.8%) was found for *S. birrea* leaf extracted with petroleum ether (0.5 mg/mL) [30]. Regarding the methanolic fraction, the highest AChE inhibitory activity was 73.8% for the *S. birrea* young stem extract [30]. On the other hand, for the dichloromethane fraction, the highest percentage inhibition was 59.9% and 59.0% for *H. caffrum* leaf and *S. birrea* young stem extracts, respectively (both at 0.5 mg/mL) [30]. In line with the results obtained for the α-amylase inhibitory activity, the extracts enriched with organic solvents, such as dichloromethane and methanol, achieved the highest inhibition of AChE.

## 3. Materials and Methods

### 3.1. Chemicals

Most of the reagents were acquired from Sigma-Aldrich (Steinheim, Germany), with the exception of Folin–Ciocalteu’s reagent, gallic acid, and catechin, which were supplied by Sigma Chemical Co. (St. Louis, MO, USA); and dimethylsulfoxide (DMSO) by AppliChem (Darmstadt, Germany).

American Type Culture Collection (ATCC, Manassas, VA, USA) provided Caco-2 clone type C2BBe1 and HT29-MTX cell lines, whereas Invitrogen Corporation (Life Technologies, S.A., Madrid, Spain) was the supplier of Dulbecco’s Modified Eagle Medium (DMEM), Hank’s Balanced Salt Solution (HBSS), Fetal Bovine Serum (FBS), non-essential amino acids, penicillin, streptomycin, and trypsin-EDTA.

### 3.2. Samples

KB leaf was collected during October 2019 in a kiwi orchard (Mini-Kiwi Farm) located in Landim, Vila Nova de Famalicão, Portugal (GPS: 41.376705, −8.471039). Leaves were removed from ten different *A. arguta* plants and subsequently dehydrated (Excalibur Food Dehydrator, USA) at 41 °C for 24 h and grounded in a miller (Moulinex A320) to obtain particles with 1 mm of size. Subsequently, samples were stored at 4 °C until extraction.

### 3.3. Microwave-assisted Extraction (MAE)

MAE was accomplished with a MARS-X 1500 W (Microwave Accelerated Reaction System for Extraction and Digestion, CEM, Mathews, NC, USA) using closed Teflon extraction vessels. Briefly, samples of dried powdered leaf (300 mg) were extracted with 10 mL of solvent (water, ethanol and ethanol:water (50%, *v/v*)) [31]. Microwave power was fixed at 300 W and the extraction was performed at selected temperatures (72–94 °C) during 1 to 5 min, with constant medium stirring, according to Benlloch-Tinoco et al. [32], with minor modifications. After extraction, samples were filtrated through a Whatman No. 1 filter paper. The aqueous extract was frozen at −80 °C for subsequent lyophilization (Telstar, model Cryodos -80, Spain), while the alcoholic extract was evaporated at 40 °C (Vaccum Controller V-800, Büchi, Switzerland). The hydroalcoholic extract was firstly evaporated and then lyophilized to eliminate the residual water. Lastly, samples were stocked at room temperature (20 °C) and covered from light until further analysis.

### 3.4. Determination of Total Phenolic and Flavonoid Content

Total phenolic content (TPC) was measured spectrophotometrically following the Folin-Ciocalteu procedure [33], with minor modifications [19]. The reaction occurred during 15 min at 45 °C and 30 min at room temperature. Subsequently, the absorbance was read at 765 nm using a Synergy HT Microplate Reader (BioTek Instruments, Inc., Winooski, VT, USA). A solution of gallic acid was used as a calibration curve (linearity range = 5–100 μg/mL; *R^2^* > 0.998). Results were expressed as mg GAE/ g dw.

Total flavonoid content (TFC) was performed using a colorimetric assay [19]. The absorbance was measured at 510 nm using the Synergy HT Microplate Reader. Catechin was used as the standard curve to obtain a correlation between sample absorbance and reference concentration (linearity range = 5–300 mg/L; *R*^2^ > 0.997). Results were expressed as mg CAE/ g dw.

### 3.5. In vitro Antioxidant Activities

#### 3.5.1. DPPH Free Radical Scavenging Assay

The DPPH free radical scavenging assay was performed according to Barros et al. [34], with minor modifications [19]. The absorbance of reduction of the DPPH radical was measured by 517 nm. Trolox was the standard used for the calibration curve (linearity range: 5–125 μg/mL; *R*^2^ > 0.991). Results were expressed as half-maximal inhibitory concentration of the radical scavenging activity (IC_50_, µg/mL).

#### 3.5.2. Ferric Reducing Antioxidant Power (FRAP) Assay

FRAP assay was performed as described by Benzie and Strain [35], with minor modifications [19]. The reaction mixture was incubated at 37 °C for 30 min and the absorbance was measured at 595 nm. The calibration curve was prepared with a solution of ferrous sulfate (FeSO_4_·7H_2_O) 1 mM as the standard (linearity range: 25–500 μM; *R*^2^ > 0.998). Results were expressed in μmol of ferrous sulfate equivalents (FSE) per gram of freeze-dried plant material (μmol FSE/ g dw).

#### 3.5.3. ABTS Radical Scavenging Activity Assay

ABTS radical scavenging activity of extracts was performed according to Re et al. [36], with minor modifications. A calibration curve was plotted with ascorbic acid as standard (linearity range: 5–100 µg/mL; *R*^2^ > 0.976) and the results were expressed in IC_50_ (µg/mL).

### 3.6. Phenolic Profile Analysis Of Kiwiberry Leaf Extracts

The qualitative characterization of KB leaf extracts was conducted using a Finnigan Surveyor series liquid chromatograph, equipped with a Thermo Finnigan (Hypersil Gold) reversed-phase C_18_ column (150 mm × 4.6 mm, 5 μm) (Merck) at 25 °C (Merck Hitachi Column Oven L-2300), following the procedure described by Fernandes et al. [37].

Detection was carried out at 280 nm. Solvents were (A) H_2_O/CH_3_COOH (99:1; *v/v*) and (B) CH_3_COOH/CH_3_CN/H_2_O (1:20:79; *v/v/v*) with the gradient 80–20% A over 55 min, 20–10% A from 55 to 70 min and 10–0% A from 70 to 90 min, at a flow rate of 0.3 mL/min. The sample injection volume was 20 μL. The chromatographic column was washed with 100% B for 10 min and then stabilized with the initial conditions for another 10 min.

The mass detector was a Finnigan LCQ DECA XP MAX (Finnigan Corp., San Jose, CA, USA) quadrupole ion trap equipped with atmospheric pressure ionization (API) source, using electrospray ionization (ESI) interface. Spectra were recorded in the negative ion mode between *m/z* 120 and 2000. The mass spectrometer was programmed to do a series of three scans: a full mass, a zoom scan of the most intense ion in the first scan, and an MS-MS of the most intense ion using relative collision energies of 30 and 60 V.

### 3.7. Reactive Species Scavenging Capacity

For the reactive species scavenging capacity assays, extracts, and standards (catechin, gallic acid and trolox) were prepared according to Pinto et al. [19].

#### 3.7.1. Superoxide Radical Scavenging Assay

The superoxide radical (O_2_^•−^) scavenging assay was performed according to Gomes et al. [38]. This colorimetric method is based on the NBT reduction into a purple colored diformazan by reaction with O_2_^•−^. The absorbance was read at 560 nm for 5 min at 37 °C. Results were expressed as the inhibition, in IC_50_, of the NBT reduction to diformazan.

#### 3.7.2. Hypochlorous Acid Scavenging Assay

The hypochlorous acid (HOCl) scavenging assay was performed according to Gomes et al. [38]. The assay was read at 528 and 485 nm, for emission and excitation wavelengths (37 °C). Catechin and gallic acid were used as positive controls. Results were expressed as the inhibition (in IC_50_) of HOCl-induced oxidation of DHR.

#### 3.7.3. Nitric Oxide Scavenging Assay

The nitric oxide (^•^NO) scavenging assay was performed according to Gomes et al. [38]. The assay was performed after incubation at 37 °C during 30 min. The absorbance was measured in the microplate reader with emission wavelength at 528 nm and excitation at 485 nm. Catechin and gallic acid were employed as positive controls. Results were expressed as the inhibition, in IC_50_, of ^•^NO-induced oxidation of DAF-2.

#### 3.7.4. Peroxyl Radical Scavenging Assay

The peroxyl radical (ROO^•^) assay was performed as stated by Ou et al. [39]. The fluorescence signal was monitored every minute at the emission wavelength at 528 nm and the excitation at 485 nm, until the total decay of fluorescence. Trolox was employed as standard control. Results were expressed as the ratio between the slope obtained for extracts or positive controls and the slope of Trolox (S_sample_/S_Trolox_) corresponding to the ROO^•^-induced oxidation of fluorescein.

### 3.8. Cell Viability Assays

The cell viability assays were performed employing two different intestinal cell lines (Caco-2 and HT29-MTX) by an MTT assay, according to Pinto et al. [19]. Passages 50–52 and 18–20 of Caco-2 clone type C2BBe1 and HT29-MTX were, respectively, used. The assay was performed as stated by de Francisco et al. [22]. DMEM was used as the positive control, while Triton X‑100 at 1% (*w/v*) was the negative control.

### 3.9. α-Amylase Activity

The *α*-amylase activity was performed on the most promising extract (considering the results obtained in the previous assays) using an Amylase Activity Assay Kit (Sigma, MAK009) and according to the manufacturer’s protocol. This assay was carried out using a coupled enzymatic method, which results in a colorimetric (405 nm) product. Nitrophenol (2 mM) was used as standard. The results were expressed as a percentage of *α*-amylase inhibition.

### 3.10. Acetylcholinesterase Activity

The AChE activity was also determined for the most promising extract (considering the results obtained in the previous assays) using an AChE activity assay kit (Sigma, MAK119) and following the manufacturer’s protocol. The colorimetric product (412 nm) was formed in proportion to the AChE activity. The calibrator (200 U/l) provided by the Kit was used as a standard. Results were expressed as percentage inhibition of AChE.

### 3.11. Statistical Analysis

Statistical analysis was performed employing IBM SPSS Statistics 26.0 software (SPSS Inc., Chicago, IL, USA). All data were reported as mean ± standard deviation of, at least, three independent assays. One-way ANOVA was applied to evaluate the differences between samples and post-hoc comparisons of the means were carried out with Tukey’s HSD test, being *p* < 0.05 accepted as denoting significance. The IC_50_ values of ROS and RNS scavenging activity were calculated using GraphPad Prism 7 software (GraphPad, La Jolla, CA, USA).

## 4. Conclusions

The results obtained in this study demonstrated that MAE is an effective technique to recover antioxidant and phenolic compounds from KB leaf extracts. The hydroalcoholic extract revealed the best results, achieving the highest antioxidant and free radical scavenging activity. The chromatographic analysis performed by HPLC-DAD-EIS-MS confirmed the presence of phenolic compounds, particularly caffeoylquinic acid, quercetin-*O*-deoxyhexoside-rutinoside, apigenin-rutinoside/Procyanidin B1-B7, kaempferol and their derivatives. On the other hand, the intestinal cell viability was not affected at concentrations up to 100 μg/mL for Caco-2 and HT29-MTX. Additionally, the hydroalcoholic extract showed a mild inhibitory activity against *α*-amylase and AChE enzymes. These results allow to conclude that KB leaf extracted with MAE is an excellent option to obtain natural antioxidants and health promoter compounds that may be used in food and nutraceutical industries. Further studies should be performed to evaluate the intestinal permeability of the extracts in order to screen the metabolites absorbed by the human intestine.

## Figures and Tables

**Table 1:** Total polyphenol content (TPC), total flavonoid content (TFC), DPPH and ABTS scavenging activity and antioxidant activity evaluated through FRAP assay in the different KB leaf extracts. Values are expressed as means ± standard deviation (*n* = 3). GAE, gallic acid equivalents. CAE, catechin equivalents.

	Aqueous	Hydroalcoholic	Alcoholic
TPC (mg GAE/g dw)	120.99 ± 9.31 ^c^	321.15 ± 11.85 ^b^	629.48 ± 16.99 ^a^
TFC (mg CAE/g dw)	62.17 ± 5.86 ^c^	114.20 ± 9.44 ^b^	136.81 ± 5.40 ^a^
DPPH (IC_50_, µg/mL)	211.14 ± 23.74 ^a^	95.22 ± 4.66 ^b^	98.16 ± 10.88 ^b^
FRAP (μmol FSE/g dw)	1801.98 ± 55.17 ^b^	3079.94 ± 305.63 ^a^	2884.39 ± 15.89 ^a^
ABTS (IC_50_, µg/mL)	219.14 ± 6.01 ^a^	131.58 ± 13.43 ^b^	142.96 ± 3.90 ^b^

Different letters (a, b, c) in the same row indicate significant differences between extracts (*p* < 0.05).

**Table 2:** Identification of the phenolic compounds from KB leaf extracts by HPLC-DAD-MS. The % area of tentatively identified compounds was determined at 280 nm.

Phenolic Compounds	RT (min)	[M − H]^−^	MS^2^	MS^3^	λ (nm)	Aqueous	Hydroalcoholic	Alcoholic
						(Area fraction %)		
**Unknown**	20.79	435.34	374.90; 357.14	326.84; 356.78; 195.05	277	0.15	0.07	0.19
**Procyanidin trimer C1, C2, EEC, T2**	23.65	865.38	695.20	542.93	310; 286	0.20	0.09	0.15
**Caffeoylquinic acid**	32.82	353.51	191.20; 179.27	93.13; 171.17	286; 325	9.06	6.06	2.88
**Caffeoylquinic acid**	34.70	353.51	191.20; 179.27	93.13; 171.17	286; 325	3.28	2.51	1.96
**Caffeoylquinic acid**	35.90	353.51	191.20; 179.27	93.13; 171.17	286; 325	5.20	3.52	2.59
**Caffeoylquinic acid (adduct)**	38.08	707.51	353.45	191.13 (−162); 179.13; 135.33	325	35.85	29.98	30.70
**Unknown**	44.93	465.61	405.06	167.13; 178.9; 224.93	280; 316	0.28	0.41	0.60
**Procyanidin trimer C1, C2, EEC, T2**	49.49	865.38	695.20	542.93	310; 286	2.30	3.11	3.49
	53.75					3.93	4.24	1.17
**Caffeoylquinic acid (adduct)**	56.11	707.51	353.45	173.13; 179; 191.20 (−162)	325	17.44	13.65	6.97
**Apigenin-rutinoside/Procyanidin B1-B7**		577.27	425.02	407.17	286; 325			
**Quercetin-pentoside-hexoside**	59.59	595.14	355.27(−240); 385.13(−210); 475.00 (−120); 505.13(−90)	235	280; shoulder 325	0.24	0.15	0.54
**Procyanidin B1-B7**	60.93	577.27	425.02	407,17	280	0.15	0.16	0.32
**Unknown**	63.44	445.59	384.93	223.00; 153.07; 205.20; 161.00	277	0.63	0.79	1.28
**Procyanidin trimer C1, C2, EEC, T2**	69.28	865.38	695.20; 739.13; 713.20; 577.27	542.93	280; 310	1.33	1.70	0.44
	71.56					1.77	1.72	0.54
**Procyanidin tetramer**	76.26	1153	983; 575; 865.0	830.93; 406.93	280; 322	1.02	1.15	1.72
**Procyanidin trimer C1, C2, EEC, T2**	79.08	865.38	695.20; 739.13; 713.20; 577.27	542.93	310; 286	0.39	0.32	0.80
**Kaempferol derivative**	92.07	781.95	285.17; 739.15	257.20	268; 346	0.69	13.34	18.01
**Quercetin-*O*-deoxyhexoside-rutinoside**	93.53	755.94	301.13	179.17	352; 256	9.79	9.48	16.04
**Quercetin-*O*-rutinoside/ Quercetin-*O*-galactoside-*O*-rhamnoside/Quercetin-*O*-rhamnosyl-galactoside**	95.41	609.27	300.16 (−309)	271.31; 179.11; 255.35; 151.16	265;352	2.79	3.82	5.22
	97.69				265; 352	3.52	3.74	4.39
**Quercetin-3-glucoside**	97.69	463.75	301.07 (−162)	271.31; 179,11; 255.35; 151.16	352	-	-	-

MS^2^ and MS^3^ are signal intensity ratios of spectra molecular ion and respective fragments.

**Table 3:** Superoxide anion radical (O_2_^•−^), hypochlorous acid (HOCl), peroxyl radical (ROO^•^), and nitric oxide (^•^NO) scavenging capacities of KB leaf extracts. Values are expressed as mean ± standard error of the mean (*n* = 4).

Reactive Species	IC_50_ (μg/mL)			
	ROS			RNS
	O2^•−^	HOCl	ROO^•^ (S_sample_/S_Trolox_) ^*^	^•^NO
Aqueous	61.50 ± 3.67 ^a^	1.94 ± 0.24 ^a,b^	0.134 ± 0.004 ^c^	1.51 ± 0.20 ^a^
Hydroalcoholic	29.10 ± 2.90 ^c^	1.87 ± 0.04 ^a,b^	0.119 ± 0.005 ^c^	1.18 ± 0.09 ^a^
Alcoholic	31.65 ± 0.23 ^c^	2.08 ± 0.08 ^a^	0.118 ± 0.009 ^c^	1.74 ± 0.23 ^a^
*Positive controls*				
Catechin	46.88 ± 2.18 ^b^	0.20 ± 0.02 ^c^	3.281 ± 0.201 ^a^	0.94 ± 0.05 ^a,b^
Gallic acid	5.73 ± 0.43 ^d^	1.41 ± 0.17 ^b^	0.863 ± 0.022 ^b^	0.16 ± 0.02 ^b^

Different letters (a, b, c, d) in the same column indicate significant differences between extracts (*p* < 0.05).

## Data Availability

Data available on request due to restrictions eg privacy or ethical.

## Sample Availability

Samples of the compounds are not available from the authors.
